# Enhanced stress-induced senescence-response may increase species lifespan

**DOI:** 10.18632/aging.203216

**Published:** 2021-06-14

**Authors:** Antonello Lorenzini, Christian Sell

**Affiliations:** 1 Department of Biomedical and Neuromotor Sciences (DIBINEM), University of Bologna, Bologna, Italy; 2 Department of Biochemistry and Molecular Biology, Drexel University College of Medicine, Philadelphia, PA 19129, USA

**Keywords:** stress, cellular senescence, DNA damage, longevity, species, lifespan

Cellular senescence is a biological phenomenon that has received many interpretations since its original description by Hayflick and Moorhead in 1961.

A hypothesis proposed over 40 years ago is now generally accepted: i.e. that cellular senescence is a regulated cellular program. For the majority of forms of senescence, the senescence program seems to be induced due to the prolonged presence of DNA damage [1]. It has also become apparent that the senescence program can develop in response to multiple internal cellular stressors such as hyper-proliferation, telomere shortening, mitochondrial dysfunction, and metabolic imbalances, as well as molecular damage caused by external insults. The scientific interpretation of the contribution of the multifaceted senescence process to health and longevity or to pathologies and aging is evolving. The current view of the beneficial aspects of senescence is that in response to developmental clues, cellular senescence participates in shaping body growth, while later during adult life senescence contributes to wound healing and serves as an intrinsic block to cancer development. Through a unique senescence associated secretory phenotype (SASP), a combination of secreted cytokines, growth factors and proteases, senescence may favor tissue repair and recruitment of immune cells. The immune cells carry out the elimination of senescent cells, thus closing the circle and restoring tissue homeostasis. In terms of tumor formation, senescence is thought to be a primary, cell intrinsic barrier for tumor formation, driving cells with oncogenic mutations into an irreversible growth arrest. In the later stages of life however, lifelong exposure to stressors may cause an overabundance of senescent cells that contribute to pathologies during aging. Such pathologies are caused both by the loss of cellular function but also disruption of the tissue microenvironment through an excessive SASP, that may contribute to the chronic inflammation characteristic of aged tissue and paradoxically also contribute to cancer development by creating a pro tumor microenvironment.

**Figure 1:**
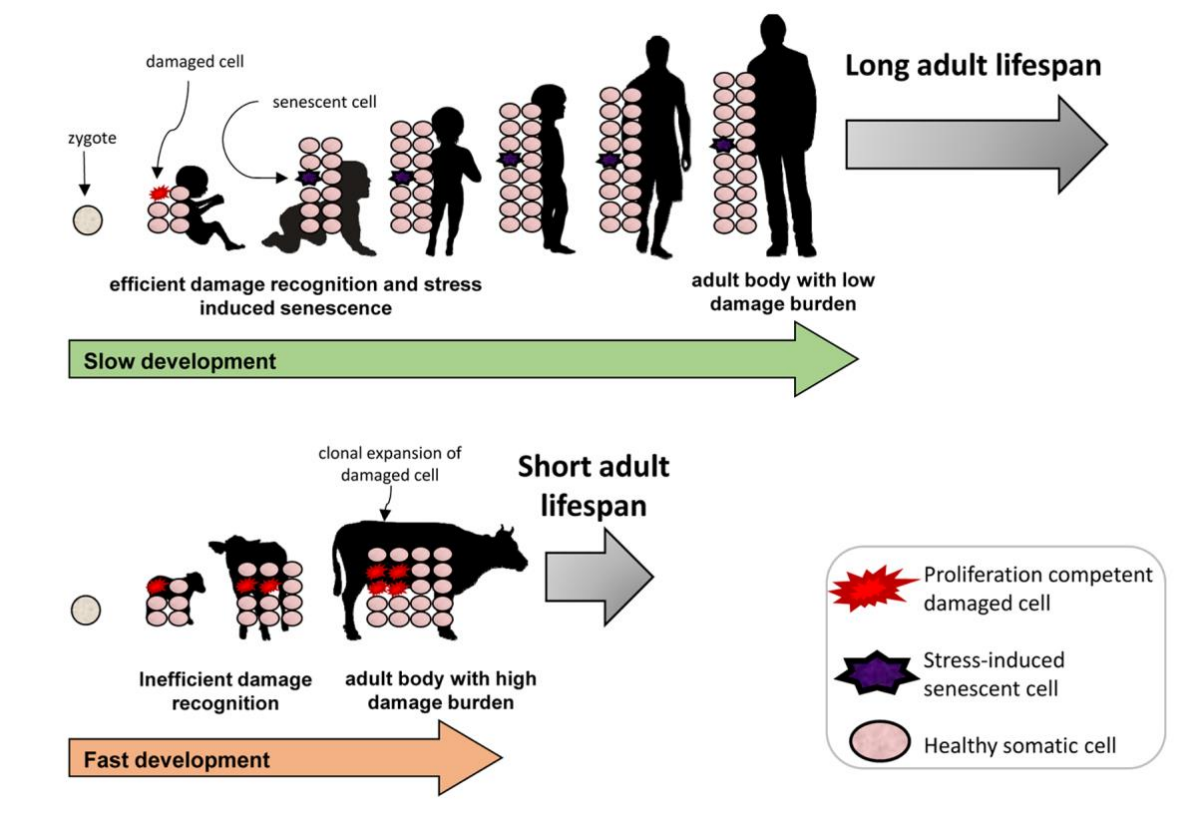
The positive effect of stress-induced senescence during development.

In this generally accepted view, cellular stress is considered a relevant senescence inducer particularly during adult and late life. In our experimental setting, we have studied the cellular response of different species to stressors, which challenge the genome directly, such as etoposide and neocarzinostatin [2,3] or indirectly such as colcemid [4]. Interestingly, we have observed that the capacity to activate the senescence program in response to genotoxic stress is more robust in cells derived from long-lived species [5]. This result is consistent with our previous work on the relative ability to recognize DNA damage in cells derived from species with differing lifespans, suggesting that long-lived species have both a greater capacity to recognize DNA damage [6] and a greater capacity to achieve a stable cell cycle arrest in response to an equivalent level of DNA damage [2]. Conceptually, this data may be reconciled with the known functions of senescence by considering senescence during development, and we propose that this enhanced ability to trigger senescence prevents clonal evolution of damaged cells [Figure 1]. This concept is analogous to the cell intrinsic anti-cancer role of senescence. Orderly induction of senescence occurs at specific sites in the embryo but senescence in response to damage must also occur. It is reasonable to predict that cells reaching a threshold level of damage will undergo senescence and that this threshold may be reduced in long-lived species. Certainly it is clear that human cells will not progress through the cell cycle in the presence of only a few DNA breaks while rodent cells are much more permissive for DNA damage in terms of cell division. Perhaps a similar situation exists with the senescence program. A species advantage may be derived from such a potential difference in senescence by reducing the number of cells with fixed mutations in the embryo. Such cells may harbor mutations, which impact cellular function in adulthood and may be silent in the undifferentiated state. If allowed to contribute to the adult organism, these cells may contribute to age-related decline in response to the stochastic aging process.

This hypothesis fits well with a concept we have developed regarding the evolution of longevity: that time constraints are a key factor in the environmental niche in which a species evolves [7]. More stringent damage recognition capacity may evolve in association with slower developmental trajectories, while more rapid development may be obtained by imposing less stringent “quality checks” at the cellular level, the loss of which leads to elevated damage at older ages and a relatively short lifespan. These considerations provide a molecular explanation for a well-described relationship that has been observed across numerous taxa: longevity is intertwined with the rate of development [8].

Thus, we propose that stress induced cellular senescence during development may be relevant to species lifespan. Clearly, we have arrived at this hypothesis very indirectly. By comparing species of different longevity in their capacity to induce apoptosis and stress induced cellular senescence [5], but we feel that the data suggest a number of potentially interesting scenarios related to the evolution of species lifespan.
